# Putting early sensory neurons to sleep

**DOI:** 10.7554/eLife.93339

**Published:** 2023-11-10

**Authors:** Julia Fadjukov, Gregory Schwartz

**Affiliations:** 1 Department of Ophthalmology, Northwestern University Evanston United States; 2 Department of Neuroscience, Feinberg School of Medicine, Northwestern University Evanston United States; 3 Department of Neurobiology, Weinberg College of Arts and Sciences, Northwestern University Evanston United States

**Keywords:** retinal ganglion cells, in vivo recordings, brain, efficient coding, anesthesia, early sensory systems, Mouse

## Abstract

Neurons that transmit information from the retina to other parts of the brain are more affected by anesthesia than previously thought.

**Related research article** Boissonnet T, Tripodi M, Asari H. 2023. Awake responses suggest inefficient dense coding in the mouse retina. *eLife* **12**:e78005. doi: 10.7554/eLife.78005.

To better understand how animals sense their environment, studies measuring brain activity need to include neural recordings in awake behaving animals (Steinmetz et al., 2018; Chorev et al., 2009). However, such experiments are often difficult to conduct, and researchers also need to consider how the stimulus is applied and account for behaviors that may impact the stimulus being measured. Active sensation, such as sniffing or eye movements, can make it challenging to control and quantify a stimulus to the extent required for many analyses (Deschênes et al., 2012; Land, 1999).

Experiments in awake animals capture more natural behavior, while those conducted in anesthetized animals or acute brain slices allow sensory stimuli to be controlled more tightly. The latter can also identify more mechanistic details of the brain structures involved in sensory processing. Thus far, it has been widely assumed that neurons behave similarly enough across these different conditions so that insights from one type of experiment can be translated to another.

However, the effects of anesthesia (and brain slicing) on various parts of the central nervous system have been a topic of considerable attention (Hao et al., 2020). For example, cognitive behaviors, like attention and motivational state, which have a large impact on neural activity, are absent in anesthetized animals (Hembrook-Short et al., 2017; Rossi et al., 2013). But neurons in early sensory systems, such as the retina, already process much of the neural code before it is even transmitted to the brain visual centers. These early sensory systems thus receive limited feedback from the brain and are thought to be less affected by anesthesia (Gastinger et al., 2006). Now, in eLife, Tom Boissonnet, Matteo Tripodi and Hiroki Asari at the EMBL Rome and the Université Grenoble Alpes report new findings that challenge this assumption (Boissonnet et al., 2023).

Boissonnet et al. recorded and compared how the output neurons of the retina (the retinal ganglion cells) responded to light in awake animals, in anesthetized animals and in experimentally isolated retinas. Activity levels of the ganglion cells in the isolated retinas were recorded through spike recordings. For recordings in living animals, Boissonnet et al. inserted electrical probes directly into the optic tract, the nerve bundle that relays visual information to the brain. Boissonnet et al. then applied full-field light modulation to stimulate the entire retina with light of equal intensity to avoid the effects of eye movements, and also accounted for pupil constriction. This allowed them to give the retina nearly identical stimuli across the three conditions.

The study revealed that ganglion cells in awake animals were able to respond to substantially higher temporal frequencies of light stimuli than ganglion cells in an animal under anesthesia or ganglion cells in isolated retinas. Isolated retinas also showed markedly lower spiking activity (Figure 1).

**Figure 1.**
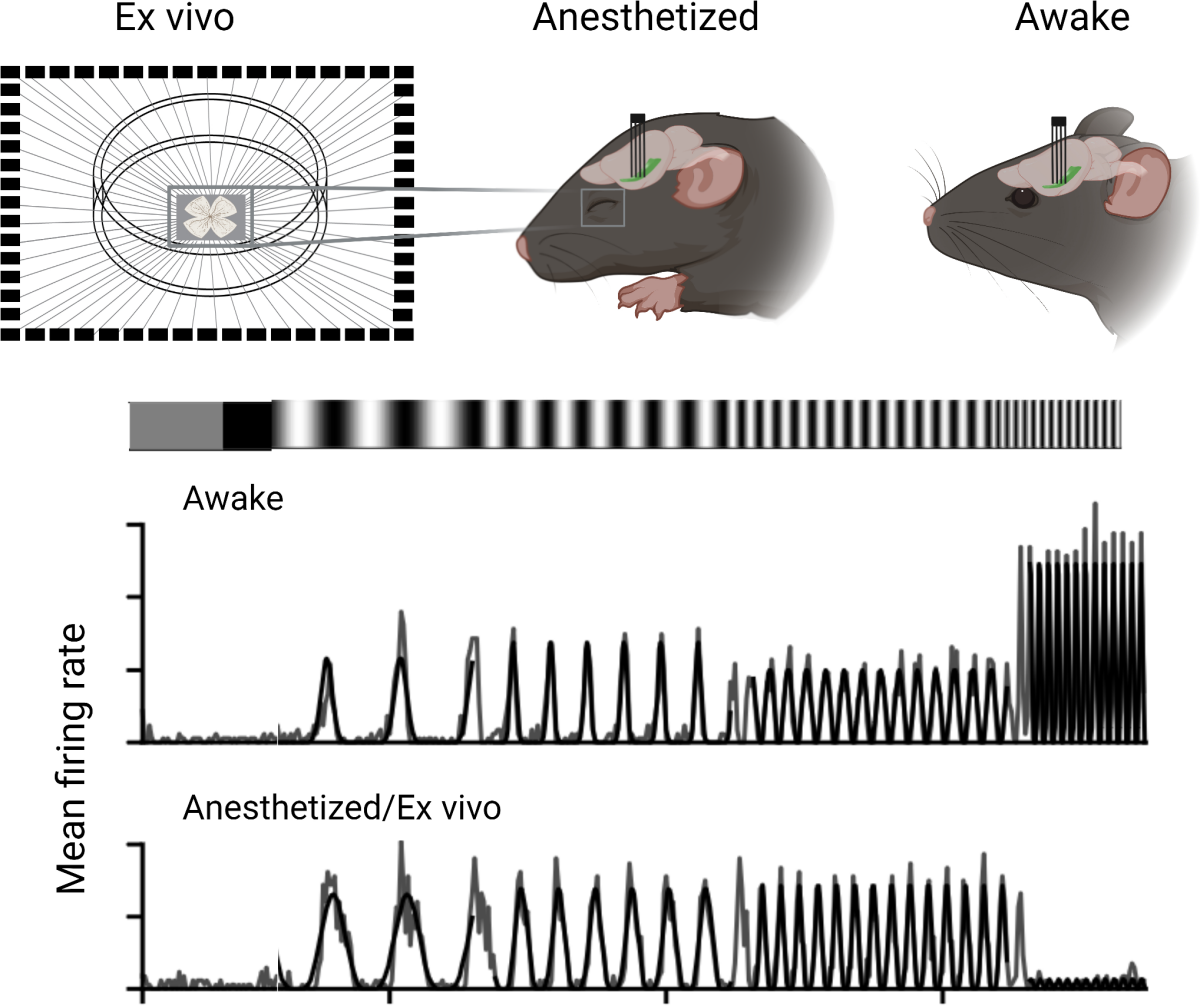
Experimental conditions affect the output of retinal ganglion cells in mice. Boisonnet et al. studied the characteristics of retinal ganglion cells in mice by comparing how they respond in awake and anesthetized animals, as well as isolated retinal cells (ex vivo). The recordings in the live animals were carried out by placing an electrode into the optic tract and measuring their neural activity when exposed to a flickering light. These results showed that awake animals had faster and stronger responses to the light stimuli compared to anesthetized and isolated preparations.

Image credit: Created using Biorender and adapted from Figure 4 of the manuscript by Boissonnet et al., 2023.

As with many innovative approaches, the study of Boissonnet et al. raises at least as many new questions as it answers. Contrary to previous hypotheses, anesthesia appears to have profound effects even at the early stages of visual processing within the retina. From a mechanistic perspective, this is, perhaps, not terribly surprising. Both anesthetics used in the study are known to interact with a set of inhibitory receptors for the neurotransmitter GABA (γ-aminobutyric acid), and they are prevalent throughout the retina (Michelson and Kozai, 2018; Bharioke et al., 2022; Yang, 2004).

Still, the magnitude of the effect – the anesthetized preparation was substantially slower than even the isolated retina – is an important point to consider when interpreting recordings in the visual system of awake and anesthetized animals. Interestingly, a higher firing activity and faster response dynamics in awake animals have also been found in the parts of the brain that the axons of the retinal ganglion primarily project onto (that is, the dorsal lateral geniculate nucleus and the superior colliculus; Durand et al., 2016; De Franceschi and Solomon, 2018). The results of Boissonnet et al. suggest that at least some of these differences originate in the retina. Comparing data from live animals and isolated cells also comes with an experimental caveat. While Boissonnet et al. did their best to replicate light conditions and temperature between the different set-ups, it is likely that some sampling biases among the over 40 types of ganglion cells in mice were different in the awake compared to the isolated retinas (Goetz et al., 2022). This makes it difficult to interpret the pooled results across the different set-ups.

While this work is a critical first step, there is still a long way to go before it is possible to measure the response of retinal ganglion cells in a natural context. Boisonnet et al. did not measure spatial or movement-related responses. Instead they ensured that the stimuli were spatially uniform, and the animals were head-fixed to minimize their eye movement. Future research could build on this method to further refine the technique and ensure the behavioral contexts of the animal match how retinal ganglion cells work in natural conditions. It will also be interesting to see if other neurons in early sensory systems, particularly those in the peripheral nervous system, like the nose and ear, have comparable results to the retina, which is part of the central nervous system (Schumacher et al., 2011). For now, though, researchers should strongly consider the implications of putting early sensory neurons to sleep.
